# Exposure to a Complex Cocktail of Environmental Endocrine-Disrupting Compounds Disturbs the Kisspeptin/GPR54 System in Ovine Hypothalamus and Pituitary Gland

**DOI:** 10.1289/ehp.0900699

**Published:** 2009-06-05

**Authors:** Michelle Bellingham, Paul A. Fowler, Maria R. Amezaga, Stewart M. Rhind, Corinne Cotinot, Beatrice Mandon-Pepin, Richard M. Sharpe, Neil P. Evans

**Affiliations:** 1 Division of Cell Sciences, Institute of Comparative Medicine, Faculty of Veterinary Medicine, University of Glasgow, Glasgow, United Kingdom; 2 Centre for Reproductive Endocrinology and Medicine, Division of Applied Medicine, Institute of Medical Sciences, University of Aberdeen, Aberdeen, United Kingdom; 3 Macaulay Institute, Craigiebuckler, Aberdeen, UK; 4 Unité de Biologie du Dévelopement et Reproduction, Institut National de la Recherche Agronomique (INRA), Jouy en Josas, France; 5 Centre for Reproductive Biology, Queen’s Medical Research Institute, Edinburgh, United Kingdom

**Keywords:** environmental chemicals, GPR54, hypothalamus, kisspeptin, pituitary, prenatal exposure, sheep

## Abstract

**Background:**

Ubiquitous environmental chemicals, including endocrine-disrupting chemicals (EDCs), are associated with declining human reproductive health, as well as an increasing incidence of cancers of the reproductive system. Verifying such links requires animal models exposed to “real-life,” environmentally relevant concentrations/mixtures of EDC, particularly *in utero*, when sensitivity to EDC exposure is maximal.

**Objectives:**

We evaluated the effects of maternal exposure to a pollutant cocktail (sewage sludge) on the ovine fetal reproductive neuroendocrine axes, particularly the kisspeptin (KiSS-1)/GPR54 (G-protein–coupled receptor 54) system.

**Methods:**

*KiSS-1*, *GPR54*, and *ER*α (estrogen receptor α) mRNA expression was quantified in control (C) and treated (T) maternal and fetal (110-day) hypothalami and pituitary glands using semiquantitative reverse transcription polymerase chain reaction, and colocalization of kisspeptin with LHβ (luteinizing hormone β) and ERα in C and T fetal pituitary glands quantified using dual-labeling immunohistochemistry.

**Results:**

Fetuses exposed *in utero* to the EDC mixture showed reduced *KiSS-1* mRNA expression across three hypothalamic regions examined (rostral, mid, and caudal) and had fewer kisspetin immunopositive cells colocalized with both LHβ and ERα in the pituitary gland. In contrast, treatment had no effect on parameters measured in the adult ewe hypothalamus or pituitary.

**Conclusions:**

This study demonstrates that the developing fetus is sensitive to real-world mixtures of environmental chemicals, which cause significant neuroendocrine alterations. The important role of kisspeptin/GPR54 in regulating puberty and adult reproduction means that *in utero* disruption of this system is likely to have long-term consequences in adulthood and represents a novel, additional pathway through which environmental chemicals perturb human reproduction.

Exposure to ubiquitous environmental chemicals can have detrimental effects on human and animal health, including immune (Rier et al. 1995; Vine et al. 2000), thyroid (Langer et al. 1998), and cognitive and motor (Colborn et al. 1993) functions. Exposure to such chemicals has also been associated with increased incidences of reproductive disorders (Dickerson and Gore 2007; Fernandez et al. 2007; Toppari et al. 1996), such as precocious puberty, early menopause, and breast cancer in females (Buck Louis et al. 2008; Kortenkamp 2006) and an increased incidence of testicular dysgenesis syndrome in males (Olesen et al. 2007; Swan et al. 2000). The chemicals present within the environment include both inorganic (e.g., heavy metals such as cadmium and lead) and organic (e.g., alkylphenols, phthalates, polychlorinated biphenyls and organochlorine pesticides) compounds, and many are termed endocrine-disrupting compounds (EDCs) due to their effects on physiologic systems. These EDCs are normally present in the environment as complex mixtures and exert their physiologic effects via a variety of mechanisms (Safe 1994; Safe et al. 2002; Wilson et al. 2008); thus, the effects of each compound can be additive, synergistic, or antagonistic (Rajapakse et al. 2002) with others present within the mixture. Investigation of the complex mechanistic interactions that might occur after exposure to such mixtures is a major emerging issue for research and risk assessment (Kortenkamp 2008). The prenatal and early life stages of development, when the fetus and young are exposed to pollutants through maternal blood and/or milk, are times when the body is particularly vulnerable to the effects of EDCs (Rhind 2005), because during this time EDCs may permanently reprogram physiologic processes influencing later health and/or reproductive function (Rhind et al. 2003).

Sewage sludge, a by-product of treatment of waste water from domestic, agricultural, and industrial sources, contains numerous organic and inorganic pollutants (Giger et al. 1984; Lind et al. 2009; Smith 1995; Stevens et al. 2003; Webber and Lesage 1989), which broadly reflect the EDC mixture to which humans are typically exposed. Sewage sludge is widely used for land remediation and as an agricultural fertilizer, including on land used for grazing by farm animals. When applied to pastures, it results in modest increases in EDC concentrations in soil and herbage (Rhind et al. 2002) and provides an ideal model to investigate the effects of “real-life” exposure to complex mixtures of environmental concentrations of chemicals/EDCs (Rajapakse et al. 2002; Rhind 2002; Smith 1995; Welshons et al. 2003). Although the observed concentrations of the individual chemicals in this model would not be predicted to pose a health risk, sheep exposed to this cocktail of pollutants exhibit altered fetal testis (Paul et al. 2005) and ovary (Fowler et al. 2008) development, altered bone density and morphology (Lind et al. 2009), and altered adult behavior (Erhard et al. 2004).

Normal reproductive function depends on hypothalamic regulation of gonadal function via secretion of gonadotropin-releasing hormone (GnRH) and the pituitary gonadotropins, luteinizing hormone (LH) and follicle-stimulating hormone (FSH), which then act on the ovaries and testes to stimulate gonadal maturation, gametogenesis, and steroidogenesis. The hypothalamo–pituitary–gonadal axis is regulated by a plethora of excitatory and inhibitory hypothalamic inputs and is highly sensitive to the organizational and activational effects of endogenous steroids during fetal and perinatal life (MacLusky and Naftolin 1981; Robinson et al. 2002). Therefore, alterations in reproductive function that manifest later in life, such as the earlier timing of puberty (Euling et al. 2008; Massart et al. 2006) or reduced fertility (Andersen et al. 2006; Carlsen et al. 1992; Skakkebaek et al. 2006), could be the result of fetal exposure to sex steroid mimetics, such as EDCs, during development of this regulatory axis.

Recent work has shown that the neuropeptide kisspeptin and its receptor G-protein–coupled receptor 54 (GPR54) are vital for the central regulation of GnRH neurosecretory activity and timing of puberty (Messager 2005; Roa et al. 2008). Kisspeptins are natural ligands for GPR54 (Kotani et al. 2001; Muir et al. 2001; Ohtaki et al. 2001). Thus, it is possible that the kisspeptin/GPR54 neurosecretory system is a target through which EDCs could perturb normal reproductive function (Navarro and Tena-Sempere 2008). This possibility is highlighted by findings showing that expression of *KiSS-1* mRNA is regulated by estradiol (Navarro et al. 2004; Smith et al. 2005a, 2005b) and that kisspeptin can act as a transsynaptic modulator for conveying estradiol feedback to GnRH neurons (Navarro and Tena-Sempere 2008).

The objective of the present study was to determine whether exposure of pregnant sheep to environmental concentrations of a mixture of EDCs, via sewage sludge used as a fertilizer, disrupts the kisspeptin/GPR54 system, as determined by examination of *KiSS-1*, *GPR54*, and *ER*α (estrogen receptor-α) mRNA expression in the adult and fetal hypothalamus and pituitary and the proportions of kisspeptin positive LH-secretory gonadotropes and estradiol-receptive gonadotropes within the fetal pituitary gland.

## Materials and Methods

### Animals

Control (C) and treated (T) pregnant ewes were maintained on pasture treated with conventional inorganic fertilizers or sewage sludge, respectively. Sewage treatment has been applied to pastures since 1997, as described in detail elsewhere (Paul et al. 2005). Animals were treated humanely and with regard for alleviation of suffering. At 110 days of gestation, maternal and fetal animals were euthanized [Schedule 1, U.K. Animals (Scientific Procedures) Act of 1986]. Hypothalami and pituitary glands were collected from mothers (seven C and eight T) and their fetuses (12 C and 17 T) immediately after euthanasia and halved. For semi-quantitative reverse transcription polymerase chain reaction studies, tissues were frozen on dry ice and stored at −80°C before later mRNA extraction and analysis. For immunohistochemical studies, hemipituitaries from fetuses (seven C and eight T) were fixed in 4% paraformaldehyde for 24 hr and then stored in 30% sucrose at 4°C until processed.

### Tissue preparation

While still frozen, maternal and fetal hypothalamic blocks were cut into three coronal slices (4 mm thick in adult tissue, 2 mm thick in fetal tissue) using external landmarks on the base of the brain as previously described (Whitelaw et al. 2009). The most rostral slice (Ros) encompassed the preoptic area (POA), the mid slice (Mid) encompassed the paraventricular nucleus, and the caudal slice (Cau) contained the arcuate nucleus (ARC). From each slice, approximately 100–200 mg of tissue was harvested for RNA extraction for each animal. Approximately 100–200 mg of tissue was also harvested from the midsagittal face of maternal and fetal pituitary glands for RNA extraction. Total RNA was extracted from each tissue using TRIzol (Invitrogen, Paisley, UK) as recommended by the manufacturers, and mRNA (200–300 ng) was then reverse transcribed using Moloney murine leukemia virus reverse transcriptase (Invitrogen), random hexamers (Promega, Southampton, UK), and RNasin (Promega) as described previously (O’Shaughnessy and Murphy 1993). Purity and quantity of mRNA and cDNA were assessed using an ND-1000 spectrophotometer (NanoDrop Technologies, Wilmington, DE, USA).

### Semiquantitative reverse transcription-polymerase chain reaction

We designed oligo-nucleotide primers and Taqman probes for the *KiSS-1*, *ER*α, and β*-actin* genes using published ovine GenBank [National Center for Biotechnology Information (NCBI) 2009] sequences DQ059506, AY033393 and U39357, respectively, and Primer Express Software (Applied Biosystems, Warrington, UK). The *GPR54* primer sequences were obtained from previously published work conducted in the rat (Seminara et al. 2003). The *GPR54* primer sequences used were 100% homologous to the NCBI ovine *GPR54* (KiSS-1 receptor) sequence (EU 272411). The Taqman probes for *KiSS-1*, *ER*α, *GPR54*, and β*-actin* were synthesized by Eurogentec (Southampton, UK) and contained FAM (6-carboxyfluorescein) as the 5′-reporter and Blackhole Quencher 1 as the 3′-quencher (sequences shown in Table 1). The qPCR reactions were performed in duplicate, and a reagent blank was included within each plate to detect contamination by genomic DNA. To validate the primers for *KiSS-1* and *GPR54*, before their use in qPCR, we initially used them in a reverse transcription PCR reaction and amplified the fragments, and then we separated the fragments by 1.5% agarose gel electrophoresis, and visualized them by ethidium bromide staining. We obtained and sequenced the PCR products of the predicted size for both genes for verification.

The qPCRs for ovine *KiSS-1*, *GPR54*, and the endogenous reference gene β*-actin* were conducted using an Mx3000P real-time PCR system (Agilent Technologies UK Ltd, Stockport, UK)) according to the manufacturer’s instructions. Each qPCR reaction (25 μL) contained PCR Buffer II [Applied Biosystems; 50 mM KCl, 10 mM Tris-HCl (pH 8.3), 1.5 mM MgCl_2_, 0.001% (vol/vol) gelatin], 0.2 mM of each deoxynucleotide triphosphate, 0.625 U AmpliTaq Gold DNA polymerase (Applied Biosystems), template cDNA (2 μL), and optimized quantities of the respective probe and primers for the genes of interest and molecular biology grade H_2_O (VWR, Lutterworth, UK). Thermal cycling conditions were 10 min at 95°C (initial denaturation step), 45 cycles of 30 sec at 95°C (denaturation step), and 1 min at 60°C (primer annealing and elongation).

We quantified *KiSS-1*, *ER*α, and *GPR54* mRNA expression using the comparative CT (cycle threshold) method (Schmittgen and Livak 2008), and we calculated gene expression relative to the reference gene (β*-actin*). Validation studies were conducted to ensure that the amplification efficiencies of the reference gene, *KiSS-1*, *ER*α, and *GPR54* were equivalent. This involved generation of relative standard curves for each gene using serial dilutions of cDNA. We plotted the resultant ΔCT values between the housekeeping gene and *KiSS-1*, *ER*α, and *GPR54* (*y*) versus log (dilution) (*x*), and calculated the slope of the line (by linear regression analysis). Slopes for *KiSS-1*, *ER*α, and *GPR54* were all < 0.1, indicating equivalent amplification efficiencies (data not shown).

### Immunohistochemistry

Frozen sections (5 μm) of fetal pituitaries were cut on a cryostat and thaw mounted onto Polysine-coated slides (VWR). We washed the cut sections from each animal (*n* = 8 C, *n* = 8 T) briefly in phosphate-buffered saline (PBS)and the sections underwent microwave antigen retrieval to unmask epitopic sites (Leong and Milios 1986; Shi et al. 1991). Briefly, we immersed the slides in 0.1 M sodium citrate buffer and heated them in a conventional 750-W microwave oven for 2 × 5 min as previously described for pituitary tissue (Franceschini et al. 2006). Slides were then left to cool to room temperature for 20 min before being washed in PBS. We blocked sections with PBS containing 10% normal goat serum (NGS) for 1 hr at room temperature and then rinsed them in PBS before addition of primary antibody. For kisspeptin and LHβ double immunolabeling, sections were incubated in a mixture of polyclonal kisspeptin antibody that was raised against bioactive residues 43–52 (kp10) of mouse metastin [1:1,000, IFR 145; Institut National de la Recherche Agronomique (INRA), Nouzilly, France] (Richard et al. 2008) and validated in ovine tissue (Franceschini et al. 2006) and a mono-clonal anti-LHβ antibody (1:10,000, generous gift from Alan McNeilly) in PBS containing 2% NGS, for 72 hr at 4°C. For KiSS-1 and ERα double immunolabeling, slides were incubated for 72 hr at 4°C with a mixture of polyclonal anti-KiSS-1 (as above, 1:1,000) and monoclonal anti-human ERα antibody (Dako UK Ltd, Ely, UK; 1:200) in PBS containing 2% NGS. After incubation with the appropriate mixture of primary antibodies, sections were then washed (3 × 5 min) in PBS and incubated for 1 hr at room temperature in a mixture of Alexafluor 488-conjugated goat anti-rabbit immunoglobulin G (IgG) and Alexafluor 594-conjugated goat anti-mouse IgG (Invitrogen, both 1:1,000 in 2% NGS). Sections were washed (3 × 5 min) in PBS before being mounted using Fluorsave mounting medium [Calbiochem (Merck), Beeston, UK] and coverslipped.

Immunohistochemical analysis of kisspeptin concentrated on expression within the pituitary. The study design did not allow collection of tissue in a manner that would allow visualization of kisspeptin immunopositive cell bodies in the hypothalamus as described by Franceschini et al. (2006).

For LHβ and ERα double immunolabeling, we sequentially immunostained sections using nickel-intensified diaminobenzidine (NiDAB) and diaminobenzidine (DAB). Briefly, sections were washed in PBS, and microwave antigen retrieval was carried out as detailed above. Sections were then incubated with 3% H_2_O_2_ in methanol for 10 min to block endogenous peroxidase activity, washed, blocked in 10% NGS in PBS (1 hr at room temperature), and incubated overnight at 4°C with monoclonal anti-human ERα antibody (Dako, 1:200 dilution) in PBS containing 2% NGS. Sections were then washed and incubated with an anti-mouse biotinylated secondary antibody (1:1,000, Dako) for 1 hr at room temperature, washed again, and positive immunostaining visualized using a Vecstatin ABC kit (Vector Laboratories, Peterborough, UK). Immunolocalization of ERα was visualized using NiDAB to produce a blue-black color. Slides were then washed and incubated with polyclonal anti-ovine LHβ (AFP 69707P, 1:10,000; Crawford et al. 2000) for 1 hr at room temperature before processing as detailed above, except that LHβ immunolocalization was visualized using DAB, to produce a brown product. Slides were then rapidly dehydrated in increasing concentrations of alcohol, mounted using DPX (Raymond Lamb, Eastbourne, UK) and coverslipped. Pituitary sections from all C and T animals were run for each antibody at the same time and under the same conditions to ensure comparability. Negative controls for all immunohistochemical procedures were included and indicated specific binding of all antibodies used.

The number of LHβ immunoreactive cells (brown cytoplasmic staining) with or without ERα nuclear immunolabeling (blue-black nucleus) and similarly the number of kisspeptin immunoreactive cells (green) that coexpressed LHβ or ERα (red) immunofluorescence were counted using a DM4000B microscope (Leica, Milton Keynes, UK). Images were captured at 40× magnification using a Leica DC480 digital camera and Leica Qwin software. Slides were evaluated subjectively but systematically by counting the number of immunodetectable cells or double-labeled cells from the same three areas for each pituitary gland (top, middle, and bottom). Because there was no difference in cell number between areas, the mean number of cells from the three areas was calculated for each pituitary and compared between C and T groups.

### Statistical analyses

All data are presented as mean ± SE. For real-time data analysis, all data were log transformed and analyzed using two-way analysis of variance. For colocalization studies, cell numbers in C and T animals were compared using Student’s *t*-tests. A *p*-value of less than 0.05 was considered statistically significant.

## Results

### Fetal KiSS-1 and GPR54 mRNA expression

**Figure 1:**
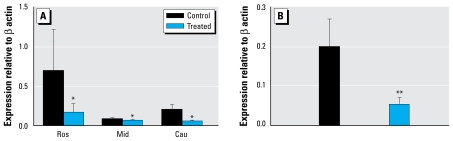
*KiSS-1* mRNA expression (mean ± SE) in fetal hypothalamus (*A*) and pituitary (*B*) after *in utero* exposure to sewage sludge chemicals (treated). *p < 0.05, and **p < 0.02 compared with respective control value.

There was no effect of sex on the level of mRNA expression for *KiSS-1* or *GPR54* in either C or T fetuses, so data were pooled. Figure 1 depicts the relative levels of *KiSS-1* mRNA expression in the hypothalamus and pituitary gland of the C and T fetuses. Hypothalamic *KiSS-1* and *GPR54* mRNA expression did not differ significantly between the areas of the hypothalamus studied. However, T fetuses expressed significantly (*p* < 0.05) less hypothalamic *KiSS-1* mRNA than did C fetuses, particularly in the Ros and Cau regions (Figure 1A). *KiSS-1* mRNA expression was also significantly lower (*p* < 0.05) in the pituitary glands of T than of C fetuses (Figure 1B). Despite the significant effects of treatment on *KiSS-1* mRNA expression, no differences were seen in either hypothalamic or pituitary gland *GPR54* mRNA expression (Ros: C, 0.14 ± 0.03; T, 0.14 ± 0.02; Mid: C, 0.60 ± 0.51; T, 0.23 ± 0.05; Cau: C, 0.14 ± 0.07; T, 0.13 ± 0.04; pituitary: C, 0.28 ± 0.086; T, 0.26 ± 0.06).

### Maternal KiSS-1 and GPR54 mRNA expression

There were no significant differences in maternal hypothalamic *KiSS-1* mRNA expression with treatment (Ros: C, 0.77 ± 0.37; T, 1.46 ± 0.79; Mid: C, 1.64 ± 0.45; T, 1.23 ± 0.76; Cau: C, 0.82 ± 0.39; T, 0.91 ± 0.91). In the maternal pituitary, *KiSS-1* mRNA expression exhibited a nonsignificant trend (*p* = 0.15) toward lower expression in T (0.42 ± 0.61) relative to relative to C (1.76 ± 0.58) animals. No significant effects of treatment were seen on *GPR54* mRNA expression in either tissue from adult ewes (Ros: C, 0.33 ± 0.2; T, 0.39 ± 0.17; Mid, C, 0.66 ± 0.54; T, 0.27 ± 0.14; Cau, C, 0.17 ± 0.03; T, 0.38 ± 0.25, pituitary: C, 0.99 ± 0.75; T, 0.70 ± 0.34).

### ERα mRNA expression

**Figure 2:**
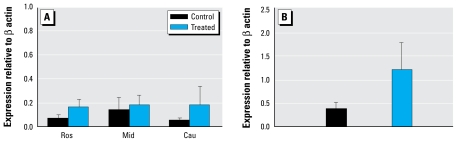
Maternal *ER*α mRNA expression (mean ± SE) in the hypothalamus (*A*) and the pituitary (*B*) of T sheep were not significantly affected by exposure to sewage sludge relative to control sheep.

Figure 2 depicts relative levels of *ER*α mRNA expression in the hypothalamus and pituitary gland of the C and T dams. T dams exhibited a trend toward higher *ER*α mRNA expression than did C dams in all areas of the hypothalamus and in the pituitary glands (Figure 2A,B). In the fetal animals, exposure to treated pastures had no effect on *ER*α mRNA expression in either the hypothalamus or the pituitary gland (Ros: C, 0.041 ± 0.025; T, 0.017 ± 0.003; Mid, C, 0.011 ± 0.003; T, 0.039 ± 0.014; Cau, C, 0.11 ± 0.10; T, 0.009 ± 0.002, pituitary: C, 0.299 ± 0.138; T, 0.246 ± 0.076).

### Kisspeptin and LHβ colocalization in the fetal pituitary

**Figure 3:**
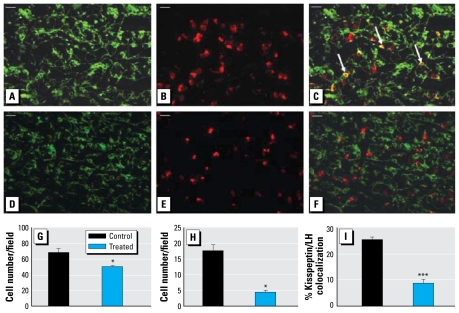
Representative photomicrographs of staining for kisspeptin (*A*, *D*) and LHβ (*B*, *E*), and merged images (*C*, *F*), in fetal pituitaries from control (top) and treated (bottom) animals. Cells showing colocalization are stained yellow (indicated by arrows). Bar = 20 μm. (*G*–*I*) Quantification per field of view of the mean LHβ-immunopositive cell number (*G*), the mean number of cells immunopositive for both LHβ and kisspeptin cells (*H*), and percentage of kisspeptin/LHβ immunopositive cells as a proportion of the total number of LHβ-immunopositive cells (*I*) in fetal pituitary tissue sections from control and treated animals. *p < 0.05, and ***p < 0.001, compared with respective control value.

Given the decrease in *KiSS-1* mRNA expression in the fetal animals, we used dual immunohistochemistry to examine whether exposure to sewage-sludge–treated pastures had any effect on the expression of kisspeptin within the fetal pituitary glands and, if so, in which cells. Figure 3 shows representative examples of the staining for LHβ and kisspeptin in the pituitary glands of C and T fetuses and the mean number of immunopositive cells observed. Exposure to sewage sludge was associated with a significant (*p* < 0.05) reduction in the number of LHβ immunopositive cells in the fetal pituitary gland (Figure 3G) and a significant reduction in the number of cells double labeled for LHβ and kisspeptin (expressed as the number of LHβ positive gonadotropes per field; *p* < 0.01; Figure 3H) or as a percentage of the total number of LHβ positive cells per field (*p*< 0.001; Figure 3I).

### Kisspeptin and ERα colocalization

**Figure 4:**
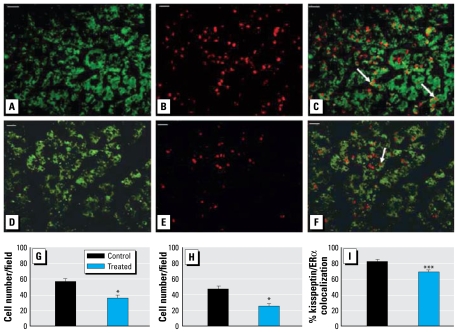
Representative photomicrographs of staining for kisspeptin (*A*, *D*) and ERα (*B*, *E*), and merged images (*C*, *F*), in fetal pituitaries from control (top) and treated (bottom) animals. Examples of cells showing colocalization are indicated by arrows. Bar = 20 μm. (*G*–*I*) Quantification per field of view of the mean ERα-immunopositive cell number (*G*), the mean kisspeptin/ERα cells immunopositive for both ERα and kisspeptin (*H*), and percentage of kisspeptin/ERα immunopositive cells as a proportion of the total number of ERα immunopositive cells (*I*) in fetal pituitary tissue sections from control and treated animals. *p < 0.01, and ***p < 0.001, compared with respective control value.

Kisspeptin expression was also analyzed relative to ERα expression in the fetal pituitary glands. Figure 4 shows representative images of kisspeptin, ERα, and double-labeled immunofluorescence and the mean number of cells expressing kisspeptin and ERα in the C and T fetuses. T fetuses exhibited significantly fewer (*p* < 0.01) ERα positive cells within the fetal pituitary glands compared with C fetuses (Figure 4G). Significantly (*p* < 0.01) fewer ERα positive cells were also present within the pituitary gland that coexpressed kisspeptin in the T relative to the C animals, expressed as both the number of cells (Figure 4H) and the percentage of colocalized cells (*p* < 0.001; Figure 4I).

### LHβ and ERα colocalization

**Figure 5:**
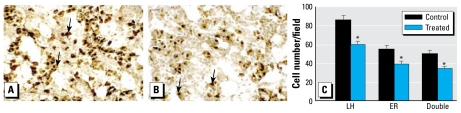
Representative photomicrographs of ERα (blue-black) and LHβ (brown) double immunohistochemistry in fetal pituitaries from control (*A*) and treated (*B*) animals. Colocalized cells are indicated by arrows; bar = 20 μm. (*C*) Quantification per field of view of the mean of LHβ, ERα, and LHβ/ERα immunopositive cells in fetal pituitary tissue sections from control and treated animals. *p < 0.05 compared with respective control value.

Figure 5, A and B, depicts representative images of pituitary tissue from C and T fetuses immunostained for LHβ and ERα, and the mean number of cells expressing LHβ or ERα or coexpressing both antigens is shown in Figure 5C. T fetuses had significantly fewer ERα positive cells (*p* < 0.01) and LHβ/ERα positive double-labeled cells (*p* < 0.005) per field (Figure 5C). However, the number of colocalized cells per field, expressed as a proportion of the total number of ERα immunopositive cells, was similar for C and T fetuses (C, 92 ± 1.7%; T, 88.7 ± 2.8%). As in the study of LHβ and kisspeptin coexpression, we observed significantly fewer (*p* < 0.01) LHβ immunopositive cells per field in T fetuses.

## Discussion

We have shown for the first time that maternal exposure to a complex mixture of chemicals/EDCs, at environmental concentrations, negatively affects the fetal kisspeptin/GPR54 neuroendocrine system. This exposure reduced the expression of both *KiSS-1* mRNA in the hypothalamus and pituitary of exposed fetuses and the proportion of kisspeptin immunopositive pituitary cells that expressed LHβ and ERα. Therefore, our results provide evidence of a mechanism through which low-level, long-term exposure to environmental EDCs may alter subsequent adult reproductive function (Navarro and Tena-Sempere 2008; Navarro et al. 2009).

This study showed that fetuses exposed to a mixture of EDCs through maternal contact with chemicals present in sewage sludge have reduced hypothalamic *KiSS-1* mRNA expression; this effect was most pronounced in the regions of the hypothalamus that encompass the POA and ARC. In both areas, kisspeptin cells coexpress ERα (Smith et al. 2005b) and in the ARC, kisspeptin immunopositive cells have been implicated in mediating estrogen negative feedback onto GnRH neurons (Franceschini et al. 2006). Therefore, EDC exposure may disturb estrogen feedback systems within the hypothalamus, which may have consequences in later life (e.g., the timing of puberty). Although this has not yet been examined in this experimental model, kisspeptin signaling is fundamental to the initiation of puberty in rodents (Clarkson and Herbison 2006; Han et al. 2005) and reproductive activity in sheep (Clarke et al. 2009; Smith et al. 2008a), and hypothalamic *KiSS-1* gene expression during puberty is significantly reduced if rats are neonatally exposed to estrogenic compounds (Navarro et al. 2009). Interestingly, despite a significant decrease in *KiSS-1* expression in our exposed fetuses, *GPR54* mRNA expression was not affected in either the hypothalamus or the pituitary gland. Although regulation of *KiSS-1* mRNA expression by estradiol is widely accepted, hormonal regulation of *GPR54* mRNA expression is not as well documented and appears to be less sensitive to steroid feedback, at least in the pituitary gland (Richard et al. 2008). Therefore, regulation of the kisspeptin/GPR54 system by estrogenic compounds is likely to be mediated primarily via the ligand rather than its receptor. Although it is not possible to determine which specific compounds were responsible for the effects noted in the T fetuses, the findings of Navarro et al. (2009) suggest that it is likely due to the activity of estrogenic EDCs known to be present in sewage sludge. Given the critical organizational events occurring *in utero*, alterations in the kisspeptin/GPR54 system could have long-term detrimental effects on adult reproductive function, and further studies will be required to investigate this possibility.

The results of the present study also indicate that maternal EDC exposure reduces fetal pituitary *KiSS-1* mRNA expression as well as lowering the proportion of gonadotropes that coexpressed kisspeptin and LHβ and the number of LHβ positive gonadotropes. Although the present experimental design did not allow us to differentiate between direct effects of EDCs at the level of the pituitary and indirect central actions, the results clearly demonstrate that maternal EDC exposure could affect fetal intrapituitary modulation of LH release. However, not all kisspeptin positive cells within the pituitary were also LHβ positive; therefore, the observed reduction in *KiSS-1* mRNA expression may also affect other pituitary cell types, including somatotropes, lactotropes, and gonadotropes that specifically synthesize FSH. Kisspeptin function within the pituitary gland, particularly during development, may not be restricted to LH release because gonadotropes are also a critical source of mitogenic and/or differentiation (Tilemans et al. 1992) and antiapoptotic factors (Melamed et al. 1999).

As with the hypothalamic kisspeptin/GPR54 system, the pituitary kisspeptin system is also sensitive to endogenous estradiol (Smith et al. 2008b). The results of this study show, again for the first time, that kisspeptin is expressed in pituitary cells that also express ERα, demonstrating that there may be important interactions between kisspeptin and estrogens in the fetal ovine pituitary. Moreover, the number of ERα positive cells, as well as the proportion of cells coexpressing ERα and kisspeptin, was significantly reduced in T fetuses. Because 92% of ERα-positive cells are also LHβ-positive gonadotropes, this reflects a significant disturbance of the interactions among kisspeptin, estrogens, and LH by chemical/EDC exposure *in utero*.

Our results extend previous work that reported the presence of kisspeptin and GPR54 in the adult pituitary gland of the rat (Gutierrez-Pascual et al. 2007; Richard et al 2008) and demonstrates for the first time that *KiSS-1* and *GPR54* mRNA and kisspeptin are expressed in the fetal ovine pituitary. The exact role played by pituitary kisspeptin and its relevance in the regulation of LH secretion (Smith et al 2008b) are presently subject to discussion. Our results indicate that kisspeptin could have paracrine/autocrine actions within the pituitary gland, and these have not yet been characterized. However, based on studies that have shown kisspeptin to be involved in LH release in the presence of GnRH (Gutierrez-Pascual et al. 2007; Navarro et al. 2005), it has been suggested that kisspeptin might act in the pituitary, in synergy with GnRH (and estradiol), as an endocrine/autocrine/paracrine signal to modulate hormone secretions from the anterior pituitary (Richard et al. 2009).

The late prenatal and early postnatal developmental stages are particularly sensitive periods of brain development with initiation of sexually dimorphic characteristics (Rhees et al. 1990). It is therefore perhaps not surprising that exposure to exogenous chemicals/EDCs in sewage sludge (Cespedes et al. 2008; Lind et al. 2009; Petrovic et al. 2002; Reddy et al. 2005) during this period has the potential to alter the kisspeptin/GPR54 systems and thus affect adult reproductive function. Under the present study conditions, the tissue levels of individual EDCs are modest (Rhind et al. 2002), so the effects seen must be due to combinations of small amounts of many different environmental chemicals (Willingham 2004). Interestingly, the adult female sheep, through which the chemical exposure occurred, were resilient to the effects of sewage sludge exposure, which supports the proposal that the effects of EDCs are more pronounced during the critical, early windows of development than in adulthood. Although the effects shown in fetuses in this study are very likely to be due to exposure of their mothers to a mixture of chemicals present in sewage sludge, identifying which particular chemicals or combination of chemicals are responsible is extremely difficult. Interpretation and, more important, extrapolation of the findings of this study to human health should be attempted with caution because it is impossible to know the exact level of human exposure and whether this is comparable to the levels of chemicals to which sheep are exposed after sewage sludge application to pasture. Although there are metabolic and physiologic differences between humans and sheep, human waste is an important contributor to sewage sludge. We therefore propose that humans themselves are exposed to many of the constituent chemicals/EDCs present in sewage sludge and in fact that the levels of some constituents in this model are probably lower than those to which humans are exposed.

We conclude that low-level exposure of fetuses to a mixture of environmental chemicals via maternal ingestion, throughout development, disrupts both the normal pattern of fetal hypothalamic and pituitary *KiSS-1* mRNA expression and also the number and proportion of kisspeptin positive cells that coexpress LHβ and ERα in the fetal pituitary. This indicates that the regulation of gonadotropes by endogenous estradiol could be disrupted. Because kisspeptin plays a major role in many aspects of reproductive axis regulation, including puberty onset, alterations to this system during development of the sexually dimorphic neuroendocrine axis are likely to affect long-term reproductive function and fertility and this will be examined in future studies of adult animals exposed to EDCs in this experimental paradigm. Humans are also exposed to a cocktail of environmental chemicals on a daily basis throughout their lives, including during development, so the kiss-peptin/GPR54 system is a potential target for environmental EDCs to alter puberty timing and reproductive function in our own species.

## Figures and Tables

**Table 1:** qPCR probes and primer sequences.

Target	mRNA primer sequence 5′–3′	Product (bp)	Reference
β*-Actin*	Forward: TCCTTCCTGGGCATGGAATC	199	U39357; NCBI 2009
	Reverse: GGGCGCGATGATCTTGATCT		
	Probe (FAM labeled): CCTTCCTTCCTGGGCATGGAATCC		
*KiSS-1*	Forward: CTGGTGCAGCGGGAGAAG	57	DQ059506;
	Reverse: GCGCAGGCCGAAGGA		NCBI 2009
	Probe (FAM labeled): ACGTGTCCGCCTACA		
*ER*α	Forward: CCCGGAAGACGTGAATCAGA	66	AY033393;
	Reverse: GTTTGCAAGGAATGCGATGA		NCBI 2009
	Probe (FAM labeled): CAGCTGGCCACCACTGGCTGC		
*GPR54*	Forward: TACATCCAGCAGGTCTCGGTG	71	Seminara et al. 2003
	Reverse: ACGTACCAGCGGTCCACACT		
	Probe (FAM labeled): CACGTGTGCCACTCTGACCGCC		
